# Supplementary material for: Comparing the effects of CETP in East Asian and European ancestries: a Mendelian randomization study
Source: Nat Commun. 2024 Jun 21;15:5302. doi: 10.1038/s41467-024-49109-z (PMC11192935; doi:10.1038/s41467-024-49109-z)
Supplement: Supplementary file 1 — Supplementary information [file 41467_2024_49109_MOESM1_ESM.pdf]

Comparing the effects of CETP in East Asian and European ancestries: a Mendelian randomization study

Dunca *et al.*

Supplementary information

Supplementary Figure 1. Regional association plots for the *CETP* locus across lipid traits (LDL-C, HDL-C, nonHDL-C, TG, TC) in the European and East Asian populations of GLGC.

Supplementary Figure 2. Mendelian randomization effect estimates of lower CETP protein concentration on cardiovascular outcomes in European population.

Supplementary Figure 3. Mendelian randomization effect estimates of lower CETP protein concentration on non-cardiovascular outcomes in European population.

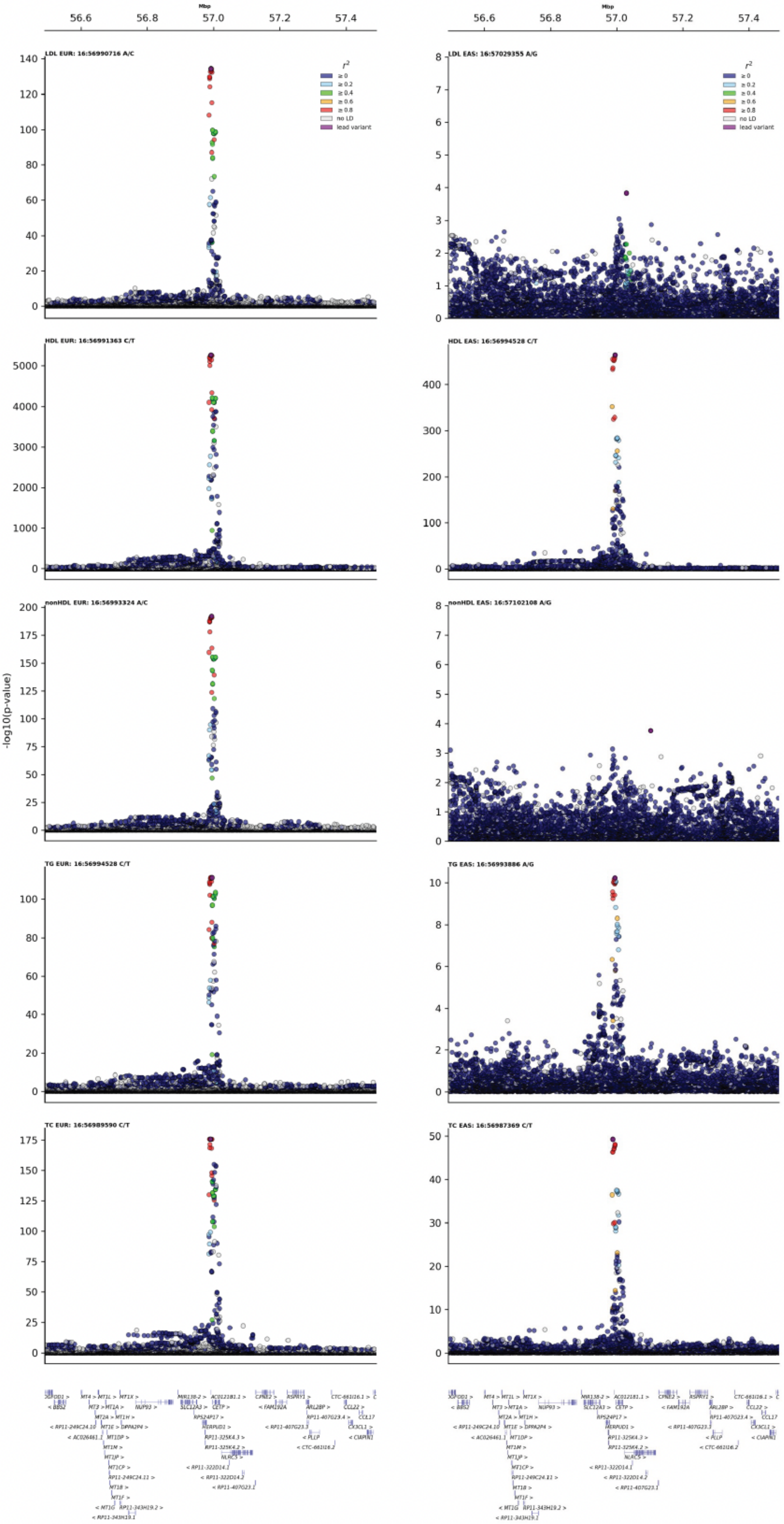

Supplementary Figure 1. Regional association plots for the *CETP* locus across lipid traits (LDL-C, HDL-C, nonHDL-C, TG, TC) in the European (left) and East Asian (right) populations of GLGC. The y-axes show the -log10(p-values) of the association between each SNP and lipid outcomes. The x-axes show the chromosomal position (GRCh37). The purple circle shows the lead SNP in each region. The colour coding indicates the linkage disequilibrium with the lead SNP based on the UK Biobank European and East Asian reference population. The source data underpinning this figure are available through figshare: https://doi.org/10.5522/04/24559537.v1 .

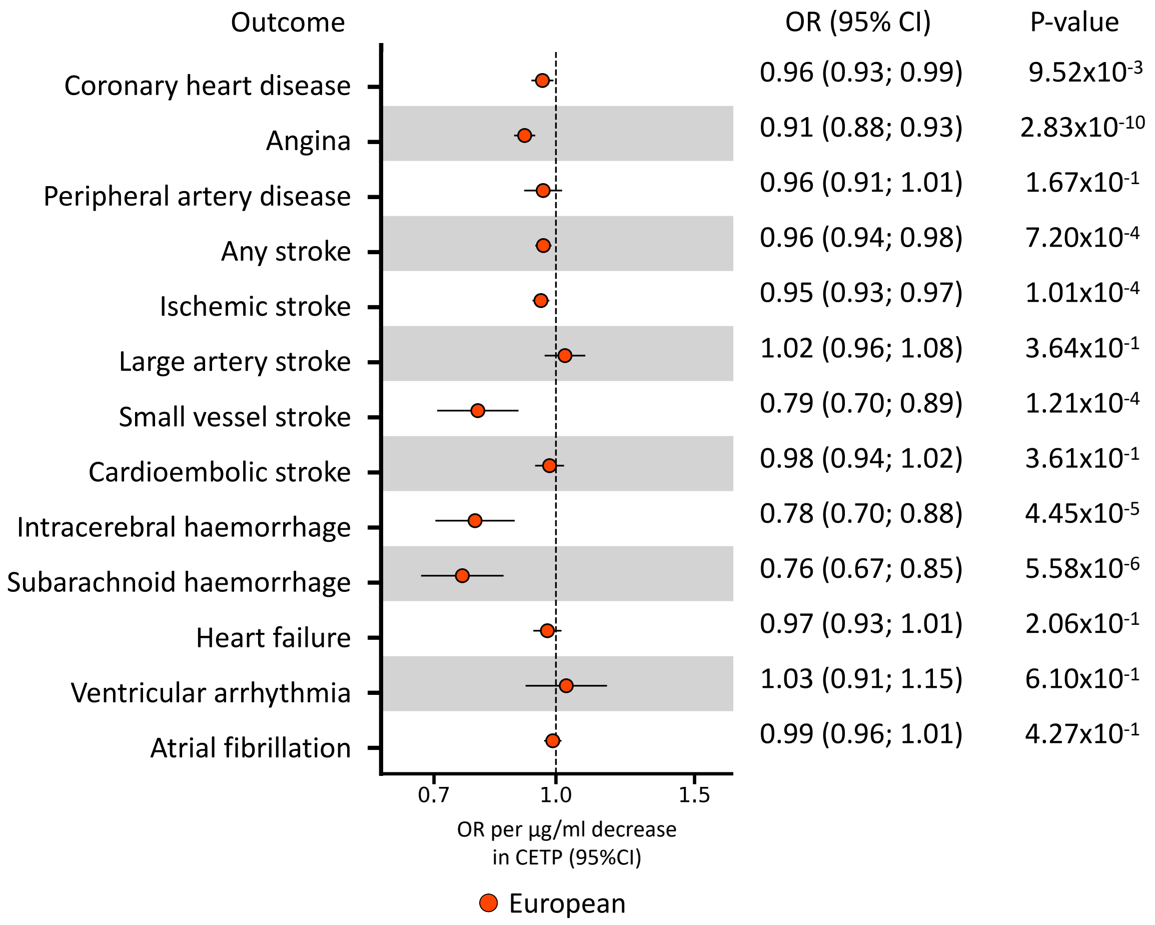

Supplementary Figure 2. Mendelian randomization effect estimates of lower CETP protein concentration on cardiovascular outcomes in European population. Effect estimates are presented as odds ratios (OR) with 95% confidence intervals (95%CI) per standard deviation increase in HDL-C (left). The OR and 95% CI are shown on the right, together with MR estimate p-value. Source data are provided as a Source Data file.

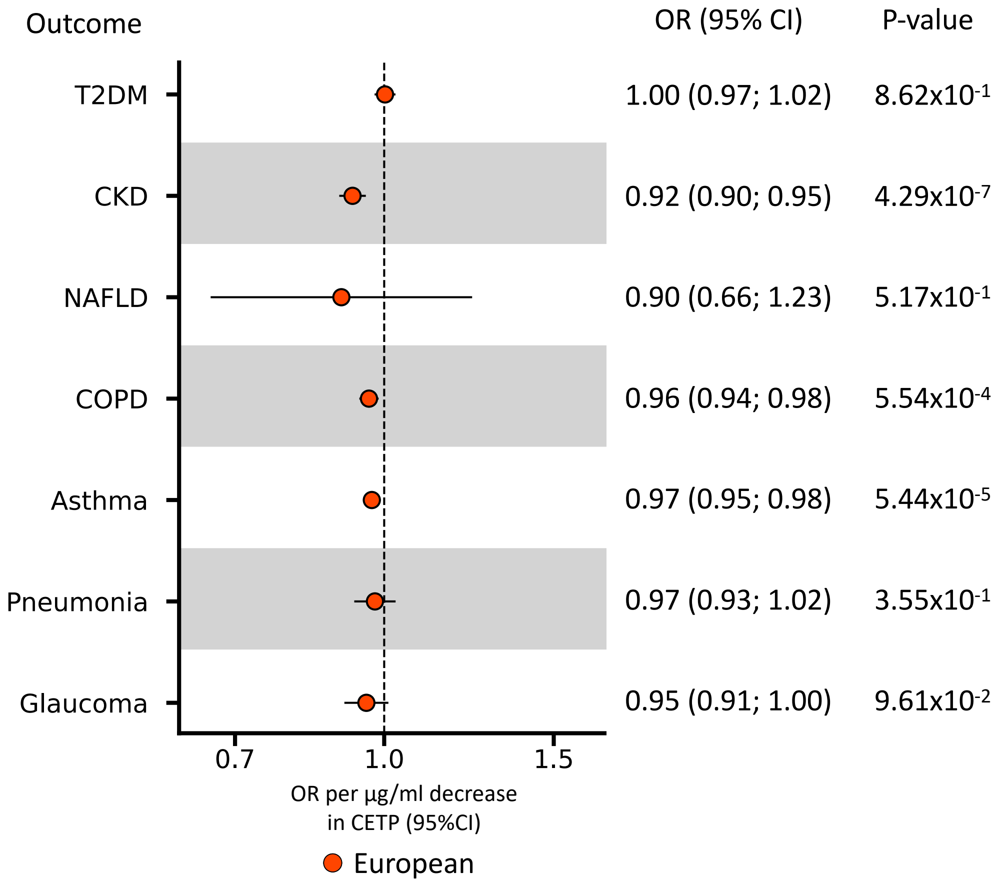

Supplementary Figure 3. Mendelian randomization effect estimates of lower CETP protein concentration on non-cardiovascular outcomes in European population. Effect estimates are presented as odds ratios (OR) with 95% confidence intervals (95%CI) per standard deviation increase in HDL-C (left). The OR and 95% CI are shown on the right, together with MR estimate p-value. T2D: Type 2 diabetes, CKD: coronary artery disease, NAFLD: non-alcoholic fatty liver disease, COPD: chronic obstructive pulmonary disease. Source data are provided as a Source Data file.
