# Supplementary material for: Comparing the effects of CETP in East Asian and European ancestries: a Mendelian randomization study
Source: Nat Commun. 2024 Jun 21;15:5302. doi: 10.1038/s41467-024-49109-z (PMC11192935; doi:10.1038/s41467-024-49109-z)
Supplement: Supplementary file 2 — Reporting Summary [file 41467_2024_49109_MOESM2_ESM.pdf]

nature portfolio

Corresponding author(s): Diana Dunca

Last updated by author(s): Apr 25, 2024

Reporting Summary

Nature Portfolio wishes to improve the reproducibility of the work that we publish. This form provides structure for consistency and transparency in reporting. For further information on Nature Portfolio policies, see our Editorial Policies and the Editorial Policy Checklist.

Statistics

For all statistical analyses, confirm that the following items are present in the figure legend, table legend, main text, or Methods section.

n/a	Confirmed	
☐	☒	The exact sample size (*n*) for each experimental group/condition, given as a discrete number and unit of measurement
☐	☒	A statement on whether measurements were taken from distinct samples or whether the same sample was measured repeatedly
☐	☒	The statistical test(s) used AND whether they are one- or two-sided<br>*Only common tests should be described solely by name; describe more complex techniques in the Methods section.*
☒	☐	A description of all covariates tested
☐	☒	A description of any assumptions or corrections, such as tests of normality and adjustment for multiple comparisons
☐	☒	A full description of the statistical parameters including central tendency (e.g. means) or other basic estimates (e.g. regression coefficient) AND variation (e.g. standard deviation) or associated estimates of uncertainty (e.g. confidence intervals)
☐	☒	For null hypothesis testing, the test statistic (e.g. *F*, *t*, *r*) with confidence intervals, effect sizes, degrees of freedom and *P* value noted<br>*Give P values as exact values whenever suitable.*
☒	☐	For Bayesian analysis, information on the choice of priors and Markov chain Monte Carlo settings
☒	☐	For hierarchical and complex designs, identification of the appropriate level for tests and full reporting of outcomes
☐	☒	Estimates of effect sizes (e.g. Cohen's *d*, Pearson's *r*), indicating how they were calculated

Our web collection on statistics for biologists contains articles on many of the points above.

Software and code

Policy information about availability of computer code

Data collection	Data were collected from publicly available GWAS repositories; no specialist software was used for this.
Data analysis	Analyses were conducted using Python v3.7.13 (for GNU Linux), Pandas v1.3.5, Numpy v1.21.6, matplotlib v3.4.3, and skyline v0.3.4a0 available through conda: https://anaconda.org/.

For manuscripts utilizing custom algorithms or software that are central to the research but not yet described in published literature, software must be made available to editors and reviewers. We strongly encourage code deposition in a community repository (e.g. GitHub). See the Nature Portfolio guidelines for submitting code & software for further information.

Data

Policy information about availability of data

All manuscripts must include a data availability statement. This statement should provide the following information, where applicable:

- Accession codes, unique identifiers, or web links for publicly available datasets
- A description of any restrictions on data availability
- For clinical datasets or third party data, please ensure that the statement adheres to our policy

The data underpinning the MR analyses are included as Supplemental 1-12, additionally the locus zoom plot data have been deposited on UCL Research Data Repository, under accession code: https://doi.org/10.5522/04/24559537.v1 . The following publicly available GWAS data (GRCh27) were sourced as exposure data: plasma CETP concentration from Blauw et al. (https://www.ahajournals.org/doi/full/10.1161/CIRCGEN.117.002034, for European ancestries only), and on HDL-C and LDL-C from GLGC (https://csg.sph.umich.edu/willer/public/glgc-lipids2021/results/ancestry_specific/, for both ancestry groups). The following GWAS were used

as source of outcome data in European ancestries: the UKB biobank analysis from Neale's lab were used as source from associations with LDL-C, Apo-B, Apo-A1, Lp [a], triglycerides, AST and ALT (http://www.nealelab.is/uk-biobank), SBP, DBP, and PP from (https://www.nature.com/articles/s41588-018-0205-x), glucose and HbA1c from (https://www.nature.com/articles/s41588-021-00852-9), CRP from (https://www.sciencedirect.com/science/article/pii/S0002929718303203), BMI from (https://www.ncbi.nlm.nih.gov/pmc/articles/PMC6298238/), CHD from CARDIoGRAMplusC4D https://www.nature.com/articles/ng.3396), asthma, pneumonia ventricular arrhythmia, intracerebral haemorrhage, angina and PAD from (https://www.nature.com/articles/s41588-021-00931-x), stroke and stroke sub-types from (https://www.nature.com/articles/s41586-022-05165-3), subarachnoid haemorrhage (https://www.nature.com/articles/s41588-020-00725-7), heart failure from (https://www.nature.com/articles/s41467-019-13690-5), type 2 diabetes from (https://www.nature.com/articles/s41588-022-01058-3), CKD from (https://www.nature.com/articles/s41588-019-0407-x), COPD from (https://www.globalbiobankmeta.org/), glaucoma from (https://www.ncbi.nlm.nih.gov/pubmed/31959993), atrial fibrillation from (https://www.nature.com/articles/s41588-018-0171-3), breast cancer from (https://www.nature.com/articles/s41588-020-0609-2), lung cancer from (https://www.nature.com/articles/ng.3892), prostate cancer form (https://www.nature.com/articles/s41588-018-0142-8), non-alcoholic fatty liver disease from (https://www.journal-of-hepatology.eu/article/S0168-8278(20)30213-0/fulltext) and eGFR from (https://www.nature.com/articles/s41588-022-01097-w). For the East Asian ancestry, the following GWAS were consulted for outcome data: Apo-B, Apo-A1, Lp[a] from (https://pan.ukbb.broadinstitute.org/), LDL-C, triglycerides, SBP, DBP, PP, glucose, HbA1c, CRP, BMI, AST, ALT, CHD, angina, PAD, ischemic stroke, intracerebral haemorrhage, subarachnoid haemorrhage, ventricular arrhythmia, type 2 diabetes, CKD, asthma, pneumonia, glaucoma, breast cancer, lung cancer and prostate cancer (https://www.nature.com/articles/s41588-021-00931-x), any stroke, heart failure, and asthma from (https://www.globalbiobankmeta.org/).

Research involving human participants, their data, or biological material

Policy information about studies with human participants or human data. See also policy information about sex, gender (identity/presentation), and sexual orientation and race, ethnicity and racism.

Reporting on sex and gender	N/A
Reporting on race, ethnicity, or other socially relevant groupings	We used the terms "East Asian/European population, individuals or ancestry " to report genetic ancestry groups
Population characteristics	The population specific characteristics for the biobanks and population cohorts used in this study are described in Supplemental Data 7.
Recruitment	N/A
Ethics oversight	N/A

Note that full information on the approval of the study protocol must also be provided in the manuscript.

Field-specific reporting

Please select the one below that is the best fit for your research. If you are not sure, read the appropriate sections before making your selection.

[x] Life sciences [ ] Behavioural & social sciences [ ] Ecological, evolutionary & environmental sciences

For a reference copy of the document with all sections, see nature.com/documents/nr-reporting-summary-flat.pdf

Life sciences study design

All studies must disclose on these points even when the disclosure is negative.

Sample size	The current study considered cardiometabolic traits with sufficiently large sample sized GWAS in both ancestries (at least 2,000 participants for quantitative traits, and at least 1,000 cases for binary traits). We did not perform any sample size calculations.
Data exclusions	To identify instruments for CETP inhibition, weighted by HDL-C or CETP plasma concentration, genetic variants outside ±50 kb of the CETP gene (Chr 16:56,995,762-57,017,757, GRCh37), with an F-statistic below 15, MAF < 0.01, and LD-clumped to an r-squared >0.3 against EUR or EAS reference populations were excluded.
Replication	The HDL-C weighted cis-MR analysis was replicated using CETP concentration (pQTLs)
Randomization	While genes cannot be randomly allocated, they are inherited in a random fashion (following Mendel's laws of inheritance).
Blinding	While subjects cannot be actively blinded from their genotype, most subjects would have been unaware of their genotype. Should they have been aware, the implication (if any) on disease would in most cases be insufficiently clear to result in any marked bias.

Reporting for specific materials, systems and methods

We require information from authors about some types of materials, experimental systems and methods used in many studies. Here, indicate whether each material, system or method listed is relevant to your study. If you are not sure if a list item applies to your research, read the appropriate section before selecting a response.

Materials & experimental systems

n/a	Involved in the study
☒	☐ Antibodies
☒	☐ Eukaryotic cell lines
☒	☐ Palaeontology and archaeology
☒	☐ Animals and other organisms
☒	☐ Clinical data
☒	☐ Dual use research of concern
☒	☐ Plants

Methods

n/a	Involved in the study
☒	☐ ChIP-seq
☒	☐ Flow cytometry
☒	☐ MRI-based neuroimaging

Plants

Seed stocks	N/A
Novel plant genotypes	N/A
Authentication	N/A
