# Supplementary material for: Comparing the effects of CETP in East Asian and European ancestries: a Mendelian randomization study
Source: Nat Commun. 2024 Jun 21;15:5302. doi: 10.1038/s41467-024-49109-z (PMC11192935; doi:10.1038/s41467-024-49109-z)
Supplement: Supplementary file 17 — Description of Additional Supplementary Files [file 41467_2024_49109_MOESM17_ESM.pdf]

Title: Supplementary Data 1.
Description: CETP cisMendelian randomisaΘon effect esΘmates on biomarkers and clinical outcomes in the East Asian and European populaΘon.

Title: Supplementary Data 2.
Description: InteracΘon test of CETP cis-Mendelian randomisaΘon effect esΘmates on biomarkers and clinical outcomes between the East Asian and European populaΘons.

Title: Supplementary Data 3.
Description: Plasma CETP concentraΘon weighted cisMendelian randomisaΘon analysis on biomarkers and clinical outcomes in the European populaΘon.

Title: Supplementary Data 4.
Description: Mean parΘcipants characterisΘcs of biomarkers across European (UKB), East Asian (this study) and Chinese ancestry groups (Millwood et al.).

Title: Supplementary Data 5.
Description: Comparison of CETP effects on clinical outcomes with Millwood et al. (scaled to 10 mg/dl) in the East Asian populaΘon.

Title: Supplementary Data 6.
Description: East Asian and European GWAS outcome datasets used in cis-Mendelian randomisaΘon analysis.

Title: Supplementary Data 7.
Description: Cohort characterisΘcs and disease definiΘon of the East Asian and European GWAS outcome datasets

Title: Supplementary Data 8.
Description: CETP HDL-C instruments selected from within and around the CETP gene in the East Asian populaΘon aŌer pruning by minor allele frequency, F staΘsΘc and clumping based on linkage disequilibrium (GRCh37).

Title: Supplementary Data 9.
Description: CETP HDL-C instruments selected from within and around the CETP gene in the European populaΘon aŌer pruning by minor allele frequency, F staΘsΘc and clumping based on linkage disequilibrium (GRCh37).

Title: Supplementary Data 10.
Description: CorrelaΘon matrix used for LD pruning in the East Asian populaΘon (GRCh37).

Title: Supplementary Data 11.
Description: CorrelaΘon matrix used for LD pruning in the European populaΘon (GRCh37).

Title: Supplementary Data 12.
Description: CETP protein concentraΘon instruments selected from within and around the CETP gene in the European populaΘon aŌer pruning by minor allele frequency, F-staΘsΘc and clumping based on linkage disequilibrium (GRCh37).

Title: Supplementary Data 13.
Description: Number of parΘcipants overlap between GLGC and outcome GWAS for East Asian and European populaΘons
